# Self-Perceived Health Status and Life Satisfaction Associated with Emotional Eating in Nursing and Medical Students: A Cross-Sectional Study in a Region of Peru

**DOI:** 10.3390/medsci13030196

**Published:** 2025-09-17

**Authors:** Jacksaint Saintila, Ana Valle-Chafloque, Luz A. Barreto-Espinoza, Elmer López-López, Norma Del Carmen Gálvez-Díaz, Isabel G. Lizarraga-De-Maguiña, Noemi Alejandrina Buenaño Cervera, Susan M. Oblitas-Guerrero, Fátima del Carmen Bernal-Corrales, Giovanna Larraín Távara

**Affiliations:** 1 Research Group for Nutrition and Healthy Behaviors, School of Medicine, Universidad Señor de Sipán, Chiclayo, Carretera a Pimentel Km 5, Lambayeque 14001, Peru; valleana2911@gmail.com (A.V.-C.); labe@uss.edu.pe (L.A.B.-E.); elmerlopez@uss.edu.pe (E.L.-L.); ncarmengd@gmail.com (N.D.C.G.-D.); lmaguinaisab@uss.edu.pe (I.G.L.-D.-M.); cerverana@uss.edu.pe (N.A.B.C.); oblitasg@uss.edu.pe (S.M.O.-G.); fatibeco@gmail.com (F.d.C.B.-C.); giovannalarraintavara@gmail.com (G.L.T.); 2 School of Nursing, Faculty of Sciences Health, Universidad Señor de Sipán, Chiclayo, Carretera a Pimentel Km 5, Lambayeque 14001, Peru

**Keywords:** emotional regulation, feeding behavior, health status, mental health, personal satisfaction, universities

## Abstract

**Background**: Emotional eating (EmE) is a maladaptive eating behavior that has been frequently observed among university students, possibly due to academic stress and lifestyle changes. However, its specific assessment in health science students has been poorly addressed, even though this population faces high levels of academic stress and emotional burden. **Objective**: This study explores the association between self-perceived health status, life satisfaction, and EmE among university students in the health field on the north coast of Peru. **Methods**: A cross-sectional study was conducted on a sample of 1213 students. Self-perceived health, life satisfaction, and EmE were assessed using validated instruments. In addition, sociodemographic data were considered as covariates and possible confounding factors. T-tests, chi-square tests, and Poisson regression with robust variance were applied. **Results**: EmE was more prevalent in women (78.0%) than in men (66.8%; *p* < 0.001). In addition, an inverse association was observed between self-perceived health and emotional eating: students with average self-perceived health (adjusted OR = 0.88; 95% CI: 0.83–0.94) and those with high self-perceived health (adjusted OR = 0.75; 95% CI: 0.69–0.81) showed a progressively lower prevalence of EmE compared to those with low self-perceived health. Similarly, high life satisfaction was associated with a lower prevalence of EmE (adjusted PR = 0.88; 95% CI: 0.80–0.96). **Conclusions**: Low self-perceived health and life dissatisfaction were significantly associated with a higher probability of EmE in medical and nursing students. These results highlight the need to strengthen university programs on mental health, emotional regulation, and subjective well-being promotion as strategies to prevent maladaptive eating behaviors in academic settings, considering gender.

## 1. Introduction

The personal, academic, and social changes that students experience during their university years can negatively influence their lifestyle habits, particularly their eating habits [1]. Emotional eating (EmE) occurs when individuals consume food under certain emotional states. Traditionally, EmE has been associated with negative emotions such as anxiety, sadness, stress, or boredom [2,3]. However, some studies have indicated that EmE can also arise in contexts of positive emotions, such as celebrations, moments of euphoria, or personal rewards, where eating fulfills a pleasant social or symbolic function [3,4]. Although eating can contribute to momentary well-being, its recurrence, whether in response to negative or positive emotions, can have negative health consequences, as in both cases it can lead to excessive consumption of high-calorie foods [5].

Both in the university context and in the general population, there has been a significant increase in unhealthy eating behaviors, with EmE being one of the most common [6,7,8]. A meta-analysis including 18 clinical surveys and 21,237 subjects reported a prevalence of 44.9% (95% CI: 29–62%) of EmE [7]. Likewise, studies conducted in different regions of the world report high percentages of students who skip meals, frequently consume ultra-processed foods, or exhibit behaviors such as EmE [1,9]. In fact, a study of Turkish nursing students found that a significant proportion resort to food as an emotional response, with women being more frequent (M ± SD: 22.68 ± 8.82 compared to men M ± SD: 17.79 ± 6.55) [6]. Also, a recent study in Romania found that 58.3% of dental students had high levels of EmE, especially women [8]. In addition, a recent study at a university in Cairo reported that final-year medical students were more susceptible to disordered eating behaviors, with a higher prevalence among women [10]. In Peru, local research has reported that at least 1 in 10 medical students exhibit some type of unhealthy eating behavior [11]. These findings consistently highlight university students, particularly those in health-related fields, as a population at higher risk of emotional eating, due to academic overload, emotional demands, and lifestyle instability commonly experienced during this stage of life.

Self-perceived health status refers to a person’s subjective assessment of their own physical and mental well-being [12]. It is a global measure obtained through a single question, usually phrased as “How would you rate your overall health?” [13]. It has high reliability and validity, as well as being an important predictor of multiple health outcomes, including morbidity and mortality [14]. In fact, it is a viable way to measure health in large-scale surveys [15]. In addition, self-rated health integrates both objective and subjective dimensions of health status, including symptoms, previous diagnoses, emotional distress, functionality, and overall well-being, making it a useful indicator in both research and public health [14,16].

Traditionally, self-perceived health status has been widely studied in the older adult population [12]; in the case of young adults, important findings were observed in university students, but in a general way [16]. However, in the specific case of medical students, the available information remains limited in scope, except for a few studies [13,15]. The analysis of self-perceived health among medical students is particularly significant, given that these students, although exposed to knowledge about health promotion, may show alarming levels of stress, anxiety, and emotional exhaustion [17]. In medical students, a perception of health negatively affected by intense academic schedules, stressful clinical rotations, and high emotional responsibility in patient care has been identified [13,18]. This negative perception of health could impact the adoption of unhealthy behaviors, such as fast food consumption, consumption of foods high in free sugars [19], or even EmE, which in turn can contribute to a progressive deterioration of overall well-being.

Few studies have focused on the association between self-rated health status and diet in medical and nursing students; most have addressed general student populations and focus on dietary intake in general [8,18,20,21]. One of these studies examines the association between the tendency to use food as an emotional coping strategy and perceived health in dental students; it identified a significant association between high EmE and self-rated health status [8]. On the other hand, another study conducted in an academic setting, although not directly evaluating EmE, reported that participants who perceived themselves as healthy had a higher frequency of breakfast consumption and showed a more positive attitude toward home-cooked meals compared to those who perceived themselves as unhealthy [21]. In addition, the findings of a study conducted in adults over the age of 19 indicated that eating breakfast, fruits, and vegetables more frequently was significantly associated with better self-rated health [20]. Although studies have examined the relationship between eating habits and self-rated health in various populations [8,20,21], specific evidence among students in academically demanding fields such as health sciences remains limited. Therefore, there is a need for further research on the specific relationships between EmE and self-rated health status in medical science students to develop interventions that promote well-being in this population.

Life satisfaction refers to the overall assessment that a person makes of the quality of their life according to their own criteria [22]. Unlike positive or negative emotions, which represent transient emotional states, life satisfaction reflects a more stable and rational cognitive assessment of how satisfied an individual is with their existence in general [22,23]. This judgment is influenced by multiple factors, such as interpersonal relationships, academic or work performance, physical and mental health, and the achievement of personal goals [24]. Previous studies have shown that university students with low levels of life satisfaction have higher levels of stress, depressive symptoms, difficulties in emotional regulation, and health risk behaviors, such as poor diet [25,26]. Specifically, health science students are no strangers to this reality. Despite being trained in areas related to healthcare, medical and nursing students are not exempt from experiencing worrying levels of dissatisfaction with life [27], which can be reflected in unhealthy eating behaviors. In fact, medical students’ life satisfaction declines during the stages of professional training [28].

Previous research has explored the role of life satisfaction in relation to different health behaviors, including EmE, particularly in university and healthcare settings [29,30,31]. Although most of these studies have not been conducted specifically on medical and nursing students, their findings allow us to identify common patterns that highlight the importance of this variable as a factor that modulates eating behavior. In fact, in a sample of university students—which did not focus exclusively on health science students—it was found that those with a greater tendency toward EmE had lower life satisfaction scores [29]. On the other hand, a study of nursing professionals found that life satisfaction was negatively associated with EmE; that is, the lower the life satisfaction, the greater the tendency to use food as an emotional coping strategy [30]. Although these results were not evident in medical students, they are particularly relevant to the present study, as they confirm this relationship in a group directly linked to medical sciences and suggest that future professionals could reflect these patterns from their formative stage if they are not adequately addressed.

Although significant relationships have been documented between self-rated health, life satisfaction, and various health behaviors in university populations and health professionals, there is a notable lack of research integrating these variables into the analysis of EmE, particularly among medical students. Most previous studies have focused on general samples of students or professionals already working in the healthcare system, leaving out those in training, such as medical and nursing students. Even so, this omission is relevant, since these students are subject to highly demanding academic conditions that can affect their perception of well-being, emotional stability, and eating patterns.

Considering that these future professionals will play a key role in promoting healthy lifestyles, it is important to understand the personal factors that can influence their own eating behaviors, especially those of an emotional nature, which often develop or intensify during college due to increased stress, autonomy, and lifestyle changes [32,33,34,35]. In response to this need, the present study aimed to analyze the association between self-rated health and life satisfaction with EmE in university students studying medicine and nursing. This approach will not only provide evidence on a poorly explored population but also offer valuable input for the design of interventions that promote comprehensive well-being from the academic training stage.

## 2. Materials and Methods

### 2.1. Design and Participants

A descriptive cross-sectional study was conducted to explore the relationship between self-rated health status and life satisfaction associated with EmE in nursing and human medicine students at a private university located on the north coast of Peru.

Participants were selected through intentional non-probability sampling. All students in both professional programs from the 1st to 7th academic year were included, as well as those enrolled in the first academic semester of 2025 (2025-I). Regular students (those enrolled in more than 12 academic credits, as defined by the Peruvian education system) were also included. However, those who did not voluntarily give informed consent and who did not provide complete data were excluded. Before beginning data collection, the sample size was calculated using the “Free Statistics Calculator” (Daniel Soper, http://www.danielsoper.com/statcalc, accessed on 20 July 2025) [36] for multiple linear regression analysis. An effect size of 0.10, a statistical power of 0.90, and a significance level (α) of 0.05 were considered. The results showed that a minimum sample of 129 students was required for the current study. However, a total of 1213 participants were surveyed, exceeding the suggested sample size. Students were invited to participate in the study via the instant messaging app WhatsApp. The survey was developed and administered using the free version of Microsoft Forms. The main objective of the study was explained to all the participants on the first page of the survey.

### 2.2. Ethical Considerations

The research protocol was reviewed and approved by the Research Ethics Committee of Señor de Sipán University (Registration code: 1279-CIEI). The question “Would you like to participate in this study?” was included on the survey’s home page, with the options “yes” or “no” as possible answers. Those who selected “no” were automatically excluded, and the survey was immediately terminated. Participation in the study was voluntary, and the confidentiality and anonymity of the data were always guaranteed. The estimated time to complete the survey was approximately seven minutes.

***Emotional eating (EmE).*** The Emotional Eater Questionnaire (EEQ) of Garaulet was used [37]. The questionnaire consists of a total of 10 questions and uses a Likert-type response scale, in which participants can choose between four options, “never,” “sometimes,” “usually,” and “always,” which correspond to the numerical values 0, 1, 2, and 3, respectively. The total score obtained for each item allows participants to be classified into three categories: non-EmE (0 to 5 points), moderate EmE (6 to 10 points), and emotional or very high EmE (11 to 30 points). For analytical purposes, participants were grouped into two categories: those with moderate EmE or very high EmE (scores ≥ 6) were grouped as “Yes,” and those without EmE (scores 0–5) as “No.” An example question from this questionnaire is “Do you have cravings for specific foods?” An evaluation of the Cronbach’s alpha coefficient of the instrument was performed using the total sample size, and a result of 0.89 was obtained. It should be noted that the questionnaire has been shown to be reliable and valid in the adolescent population in previous studies, with a Cronbach’s alpha of 0.71 [38].

***Self-perceived health status item:*** The variable was assessed using a single question: *“Overall, would you say your health is…?”*, with response options on a scale from 1 (excellent) to 5 (poor). Despite being a single item, several studies have demonstrated its validity as a subjective indicator, as well as its predictive capacity in relation to multiple physical and mental health outcomes [13,14,15].

***Satisfaction with Life Scale (SWLS):*** To measure life satisfaction, the Satisfaction with Life Scale (SWLS) proposed by Diener et al. was used [22,23]. This scale was translated and validated into Spanish by Atienza et al. [39], and then adapted to the Peruvian context by Caycho-Rodríguez et al. [40]. The SWLS is a brief measure consisting of five items that assess a person’s level of satisfaction with life. The items are statements to which participants respond using a five-point Likert scale ranging from (1) “strongly disagree” to (5) “strongly agree.” The reliability of the SWLS is adequate, with a Cronbach’s alpha of 0.76 (95% CI: 0.72–0.78) [40].

### 2.3. Covariates and Confounders

Sociodemographic information was collected from participants, which included sex (female or male), age in years (recorded as a continuous variable and subsequently categorized as under 30 years or 30 years or older), country of origin (Peru or foreign), marital status (single or not single), and highest level of education attained by parents (basic education, technical education, or university education). These variables were considered as possible covariates or confounding factors in the analyses to adjust for their possible effects on the association between the main independent variables and EmE.

### 2.4. Statistical Analysis

The analysis was performed using RStudio version 4. Descriptive analysis consisted of calculating frequencies for categorical variables and measures of central tendency and dispersion for numerical variables. Characteristics were compared according to the presence of EmE. We used chi-square tests of independence for categorical variables and the Wilcoxon rank sum test for numerical variables. Finally, Poisson regression was conducted to calculate prevalence ratios with 95% confidence intervals, assessing the association between independent variables and EmE. The regression was both simple and adjusted, including potential confounding variables such as sex, age group, parental marital status, and parental education. A *p*-value less than 0.05 was considered statistically significant.

## 3. Results

### 3.1. General Characteristics

We analyzed data from 1213 participants; 83.6% were female, and the average age was 22.7 ± 4.6 years. Ninety-one-point eight percent of participants were under 30 years of age, and 94.5% were from Peru. Most were single and had a college education. Regarding self-perceived health, most participants reported high levels. A total of 61.8% reported high life satisfaction. The prevalence of EmE (considered mild and moderate) was 76.2% (Table 1).

### 3.2. Characteristics According to EmE

When comparing characteristics according to EmE, we observed some differences. Females reported EmE more frequently (78.0%) than males (66.8%). We also observed that the frequency of EmE decreased as self-perceived health improved. About life satisfaction, those with high levels had the lowest frequency of EmE compared to participants with medium or low levels of satisfaction (Table 2).

In the simple regression analysis (Table 3), we observed average self-perceived health (PR, 0.88; 95% CI, 0.82–0.93) and high self-perceived health (PR, 0.74; 95% CI, 0.69–0.81. Those with high life satisfaction (PR, 0.89; 95% CI, 0.81–0.97) had a lower prevalence of EmE than those with low life satisfaction. In the adjusted analysis, the association remained. Specifically, those with medium or high self-rated health had 12% (PR, 0.88; 95% CI, 0.83–0.94) and 25% (PR, 0.75; 95% CI, 0.69–0.81 Those with high life satisfaction had 12% (OR, 0.88; 95% CI, 0.80–0.96) less EmE compared to those with low satisfaction.

## 4. Discussion

EmE is particularly relevant in young populations under high academic pressure, such as medical students, who not only face constant pressure but also have a future responsibility to promote healthy lifestyles [41]. This study explored the association between self-perceived health status, life satisfaction, and eating habits among nursing and human medicine students. The main findings of the study revealed a significant inverse association between self-perceived health and emotional eating, with a clear trend showing that better perceived health is associated with lower levels of EmE. The greater the perception of good health, the lower the prevalence of EmE, even after adjusting for sociodemographic variables. In addition, high life satisfaction shows an inverse association with EmE.

Sex has been identified as an important determinant in the adoption of healthy eating behaviors and in the way emotions are managed through food [42]. In the present study, the prevalence of EmE was significantly higher in women than in men, which is consistent with previous evidence indicating a greater tendency among women to use food as a strategy for emotional regulation [6]. The reasons behind this difference could be due to the fact that women are more sensitive to stress, as well as hormonal fluctuations linked to the menstrual cycle, which have been associated with changes in appetite and emotional regulation [43]. In addition, women tend to report higher levels of negative affect and often face greater social pressures related to body image [44], which can lead to a cycle of food restriction and compensation that encourages EmE [45,46].

Furthermore, the current study found a significant inverse association between self-perceived health and EmE. Specifically, students who reported average and high self-perceived health had a lower prevalence of EmE compared to those who reported poor health; this association remained significant after adjusting for sociodemographic variables. These findings suggest that a positive subjective perception of health status may act as a protective factor against maladaptive eating behaviors such as EmE.

These results are consistent with previous studies that have documented that perceived health is associated with unhealthy eating behaviors [8,18,20,21]. A study of university students reported that those with negative EmE had significantly lower self-perceived health scores [29]. Similarly, in another study, those who reported better self-perceived health were also more likely to maintain healthier eating habits, such as valuing home-cooked meals [21], which is associated with greater emotional stability and better dietary practices [47]. Although this study was conducted on secondary school students, its findings reinforce the fact that a positive perception of health status could lead to better dietary habits [21]. It should be noted that self-perceived health encompasses not only physical aspects, but also mental and emotional components [48]. Therefore, students with a negative perception of their health are likely to also experience higher levels of psychological distress or stress, which increases their predisposition to use food as a form of emotional regulation. In this regard, a recent study conducted on university students in Saudi Arabia found that high levels of perceived stress were significantly associated with lower adherence to healthy eating patterns and higher levels of EmE [49]. One possible explanation could be that poor self-perceived health could reflect a deterioration in emotional balance [48], which can lead to the use of food as an immediate source of relief [50]. In addition, chronic stress and negative emotional states activate neuroendocrine mechanisms—such as the hypothalamic–pituitary–adrenal axis—that promote the search for foods rich in sugars and fats [51], contributing to the cycle of EmE [5].

Finally, the current study found that high life satisfaction is inversely and significantly associated with EmE, even after adjusting for sociodemographic variables. In other words, students who reported higher life satisfaction were less likely to engage in EmE behaviors compared to those with low levels of satisfaction. This finding is consistent with the results of previous studies that have highlighted the role of life satisfaction in eating behavior and confirmed that emotional well-being is a relevant variable in the prevention of maladaptive eating patterns [29]. This aligns with the findings of a study conducted on nursing staff, which found a significant negative correlation between life satisfaction and EmE [30]. Similarly, the study by Mitri et al. [52] showed that depression and high stress—both closely related to low life satisfaction—significantly increased the likelihood of EmE, especially among women; however, perceived stress showed no significant association. Although these studies were conducted among healthcare professionals rather than health science students, their findings reinforce the evidence that subjective emotional state directly affects dietary decisions. High life satisfaction may promote greater emotional resilience [53,54] and better emotional regulation skills [55,56], which could reduce the need to turn to food to cope with distress [6,56]. On the contrary, those with low satisfaction may experience a disconnect between their personal goals and their everyday reality, favoring states of emotional dysregulation that lead to episodes of EmE, particularly in the face of negative emotions [6,56]. Furthermore, this pattern may be reinforced by contextual factors such as academic workload, social isolation, or lack of support, all of which are associated with lower subjective well-being in university students.

### 4.1. Limitations and Future Perspectives

This study has some limitations. For example, some relevant psychological variables such as perceived stress, anxiety, or impulsiveness were not included and could have provided a more comprehensive understanding of the phenomenon of EmE. Furthermore, the sample consisted exclusively of nursing and medical students from a single university, which may limit the generalizability of the findings to other student populations or academic contexts. In addition, there was a notable gender imbalance in the sample (83.6% female), which reflects the demographic reality of Nursing and Human Medicine programs in Peru. However, this may also limit the extrapolation of the findings to more gender-balanced populations. Future studies should consider larger and more diverse samples, as well as longitudinal designs that allow for the analysis of the temporal evolution of eating behaviors in relation to psychological well-being. On the other hand, due to its cross-sectional design, it is not possible to draw causal conclusions between the variables studied. The association observed between self-perceived health, life satisfaction, and EmE should be interpreted as correlational. Furthermore, the information was collected through self-reports, which may be subject to social desirability bias or memory errors. Finally, future research could develop and evaluate interventions aimed at improving subjective well-being and reducing EmE in educational settings, integrating personalized and gender-sensitive approaches.

### 4.2. Public Health and Practical Implications

Despite the limitations of the study, we believe that the findings may be important for implementing public health strategies targeting university populations, particularly medical students, who are not only exposed to high levels of emotional stress but will also be future professionals responsible for promoting healthy lifestyles and caring for the health of their patients. Firstly, given the results found, there is a clear need to address emotional well-being as a central component in promoting nutritional health, more specifically in preventing EmE. Secondly, university institutions should implement multidisciplinary interventions that bring together professionals such as psychologists and nutritionists, integrating psychological support, stress management, and nutrition education. The aim is to reduce the prevalence of EmE and its associated risks, such as weight gain and the adoption of unhealthy eating patterns.

Thirdly, addressing the problem with the consideration of sex is particularly important, as the findings indicate a greater vulnerability among women. This highlights the need to design interventions specifically targeted at female students. Such interventions should consider the unique emotional and social pressures faced by women in academic settings. Finally, another task to be addressed by universities is to incorporate regular assessments of EmE patterns, perceived stress levels, and life satisfaction into university health services—including psychological care, nutritional counseling, healthy habit promotion programs, and emotional support spaces—which would allow for early identification of at-risk students and provide them with timely support. It is important to consider that emotional resilience and subjective well-being in medical students can not only have a positive impact on their personal health, but are also essential for training professionals who will be tasked with encouraging healthy behaviors and psycho-emotional balance in the population through their communities.

## 5. Conclusions

The findings of this study indicate that poorer self-perceived health and lower life satisfaction are significantly associated with a higher prevalence of emotional eating (EmE), with a clear dose–response relationship observed particularly in the case of subjective health. Furthermore, women were found to be more prone to this type of maladaptive eating behavior. These results underscore the importance of promoting psychological well-being and self-care as central components in university-based interventions to prevent EmE, especially among health sciences students.

In addition, these findings emphasize the relevance of incorporating a gender-sensitive approach in the development of mental health and nutritional education programs. Future research should explore longitudinal relationships and examine the effectiveness of tailored interventions aimed at reducing EmE and improving the overall quality of life in university populations.

## Figures and Tables

**Table 1:** General characteristics of participants.

Characteristic	*N* = 1213
Sex	
Female	1014 (83.6%)
Male	199 (16.4%)
Age (years) (M ± SD)	22.7 (4.6)
Age range (years)	
<30	1114 (91.8%)
30+	99 (8.2%)
Country of origin	
Peru	1146 (94.5%)
Foreign	67 (5.5%)
Marital status	
Single	703 (58.0%)
Living with partner	510 (42.0%)
Educational level of parents	
Basic	259 (21.4%)
Technical	169 (13.9%)
University	785 (64.7%)
Self-rated health	
Low	207 (17.1%)
Medium	485 (40.0%)
High	521 (43.0%)
Life satisfaction score (M ± SD)	25.3 (5.8)
Life satisfaction	
Low	161 (13.3%)
Medium	302 (24.9%)
High	750 (61.8%)
EmE score (M ± SD)	10.3 (6.3)
EmE	
No	289 (23.8%)
Yes	924 (76.2%)

**Note.** M = mean; SD = standard deviation. EmE = emotional eating.

**Table 2:** General characteristics of participants according to the EmE variable.

	EmE		
Characteristic	No *N* = 289	Yes *N* = 924	*p*-Value *
**Sex**			<0.001
Female	223 (22.0%)	791 (78.0%)	
Male	66 (33.2%)	133 (66.8%)	
**Age (years) (M ± SD)**	22.9 (4.8)	22.6 (4.5)	0.718
**Age range (years)**			0.552
<30	263 (23.6%)	851 (76.4%)	
30+	26 (26.3%)	73 (73.7%)	
**Country of origin**			0.242
Peru	277 (24.2%)	869 (75.8%)	
Foreign	12 (17.9%)	55 (82.1%)	
**Marital status**			0.374
Single	174 (24.8%)	529 (75.2%)	
Living with partner	115 (22.5%)	395 (77.5%)	
**Educational level of parents**			0.186
Basic	66 (25.5%)	193 (74.5%)	
Technical	31 (18.3%)	138 (81.7%)	
University	192 (24.5%)	593 (75.5%)	
**Self-rated health**			<0.001
Low	19 (9.2%)	188 (90.8%)	
Medium	99 (20.4%)	386 (79.6%)	
High	171 (32.8%)	350 (67.2%)	
**Life satisfaction score (M ± SD)**	26.0 ± 6.3	25.1 ± 5.7	<0.001
**Life satisfaction**			<0.001
Low	31 (19.3%)	130 (80.7%)	
Medium	46 (15.2%)	256 (84.8%)	
High	212 (28.3%)	538 (71.7%)	

**Note.** * Chi-square test of independence; Student’s *t*-test. M = mean; SD = standard deviation; EmE = emotional eating.

**Table 3:** Poisson regression analysis of the association between self-rated health, life satisfaction, and EmE.

	Crude Regression			Adjusted Regression *		
Variable	PR	95% CI	*p*-Value	PR	95% CI	*p*-Value
**Self-rated health**						
Low						
Medium	0.88	0.82–0.93	<0.001	0.88	0.83–0.94	<0.001
High	0.74	0.69–0.80	<0.001	0.75	0.69–0.81	<0.001
**Life satisfaction**						
Low						
Medium	1.05	0.96–1.15	0.286	1.03	0.95–1.13	0.474
High	0.89	0.81–0.97	0.008	0.88	0.80–0.96	0.003

**Note.** * Adjusted for age, age group, sex, parental marital status, and parental education; PR = prevalence ratio; 95% CI = 95% confidence interval. Test: Poisson regression with robust variance.

## Data Availability

The data presented in this study are available on request from the corresponding author. The data are not publicly available due to privacy and ethical concerns.

## Abbreviations

EmE	Emotional eating
